# Supplementary material for: Key Aroma Compounds of Dark Chocolates Differing in Organoleptic Properties: A GC-O Comparative Study
Source: Molecules. 2020 Apr 15;25(8):1809. doi: 10.3390/molecules25081809 (PMC7221797; doi:10.3390/molecules25081809)
Supplement: Supplementary file 1 [file molecules-25-01809-s001.pdf]

Key aroma compounds of dark chocolates differing in organoleptic properties. A GC-Olfactometry comparative study

Zoé Deuscher [1,2], Karine Gourrat [1,3], Marie Repoux [4], Renaud Boulanger [2], Hélène Labouré [1], and Jean-Luc Le Quéré [1,*]

[1] Centre des Sciences du Goût et de l'Alimentation (CSGA), AgroSup Dijon, CNRS, INRAE, Université de Bourgogne Franche-Comté, F-21000 Dijon, France

[2] Qualisud, Univ Montpellier, CIRAD, Montpellier SupAgro, Univ d'Avignon, Univ de La Réunion,, F-34000 Montpellier, France

[3] ChemoSens Platform, CSGA, F-21000 Dijon, France

[4] Valrhona, 14 av. du Président Roosevelet , F-26602 Tain l'Hermitage, France

* Correspondence: jean-luc.le-quere@inrae.fr

Figure S1

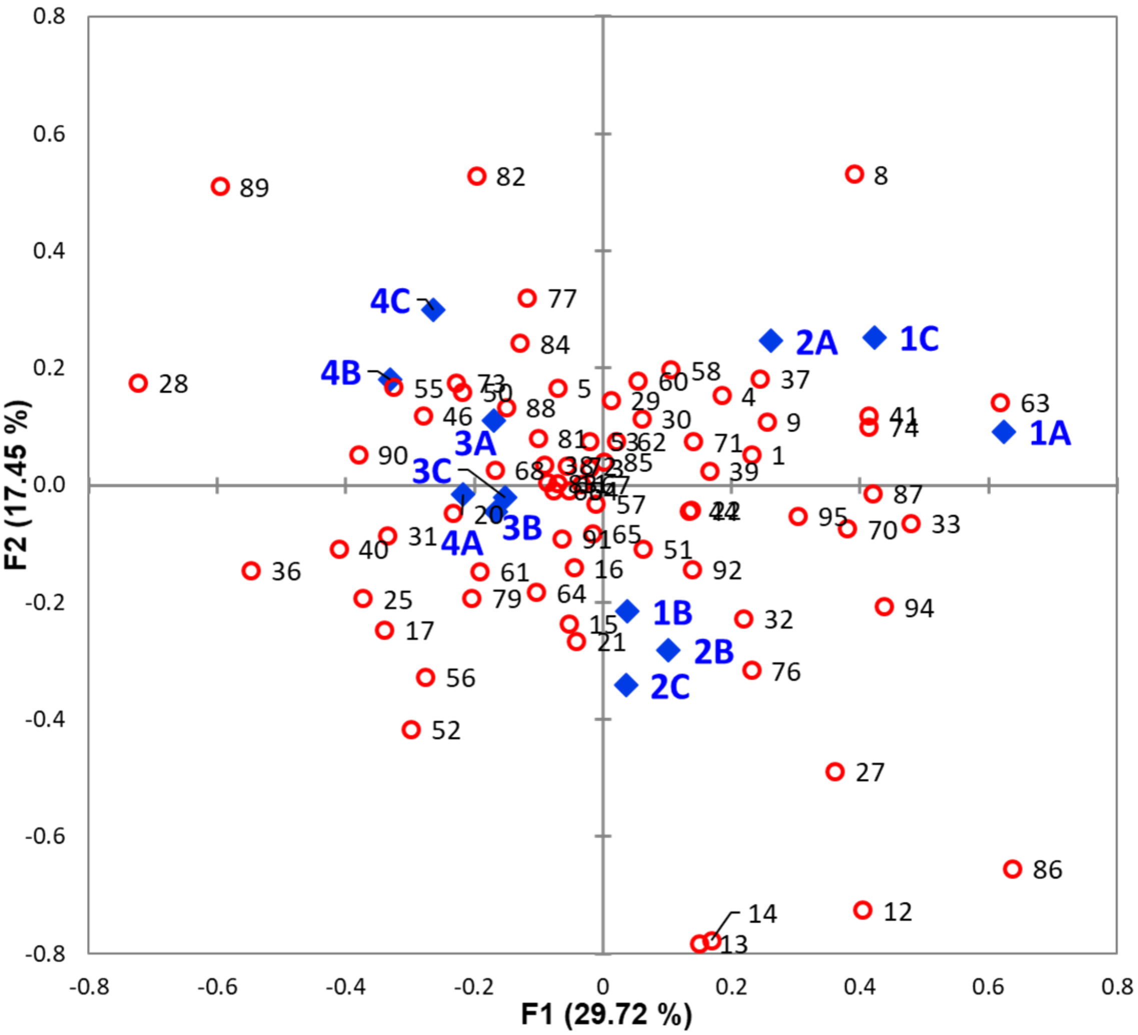

Figure S1. Correspondence analysis (CA) between the 12 samples and the 73 discriminant OAs defined by their NIF values using a NIF difference threshold >30%. OAs (light red circles) are plotted according to their NIF in samples (plain blue diamonds) in the dimensions 1 and 2 (cumulative inertia: 47.17%). The OA numbers are those found in Table 1. CA independence test: Khi² = 8540 (critical value 859, α = 0.05, degrees of freedom = 792), p < 0.0001.

Figure S2

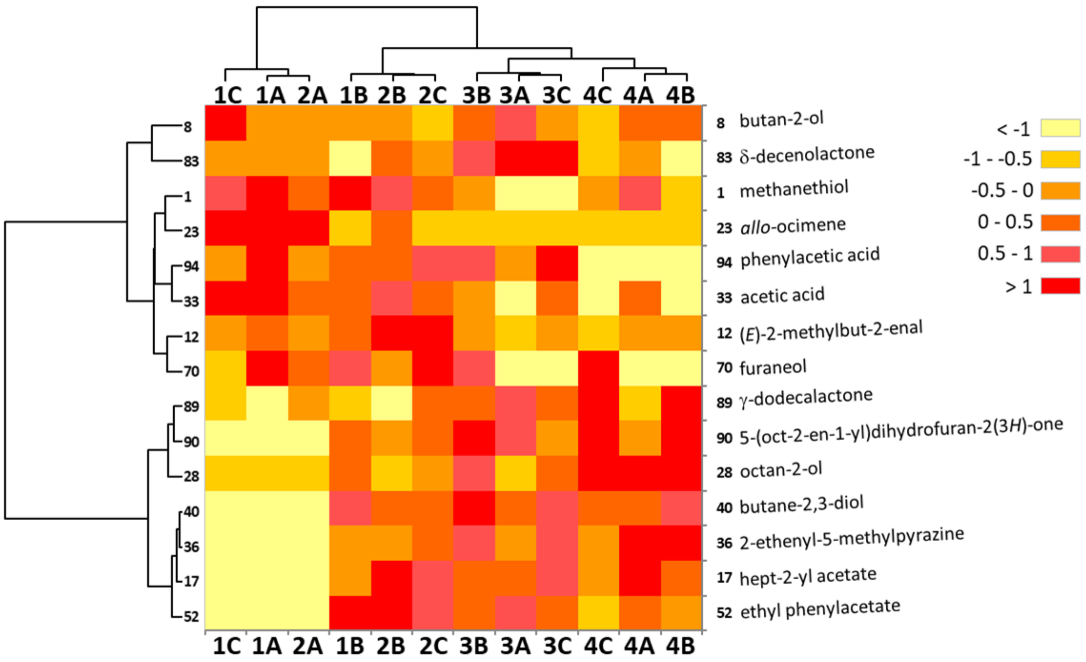

Figure S2. Heatmap displaying the results of a hierarchical cluster analysis (HCA) conducted independently on both samples and variables (OAs) dimensions, for the 31 "discriminant" OAs (see discussion). NIF values importance varies from >1 (highest value, in red) to < - 1 (lowest values, in yellow). OA numbers are those found in Table 1. The data were centered and scaled; dissimilarity Euclidian distances were used with the Ward amalgamation method; %(std) non-specific filtering was used with a 50% threshold, resulting in the display of the only 15 variables with the highest std.
